# Several antiplatelet gene SNPs, their haplotypes and *G* × *E* interactions on premature coronary artery disease

**DOI:** 10.3389/fcvm.2026.1875319

**Published:** 2026-06-29

**Authors:** Zhong-Hai Bi, Si-Cong Liu, Ying-Hong Weng, Ji-Yi Chen, Si-Yao Li, Xiao-Qun Lin, Yan-Li Liu, Liu Miao

**Affiliations:** 1 Department of Cardiology, Liuzhou People’s Hospital, Affiliated of Guangxi Medical University, Liuzhou, Guangxi, China; 2 The Key Laboratory of Coronary Atherosclerotic Disease Prevention and Treatment of Liuzhou, Liuzhou, China; 3 Department of Traditional Chinese Medicine, Liuzhou People’s Hospital, Affiliated of Guangxi Medical University, Liuzhou, Guangxi, China

**Keywords:** premature coronary artery disease, antiplatelet drugs, gene polymorphism, *CYP2C19*, haplotype, gene-environment interaction

## Abstract

**Objective:**

To investigate the impact of single nucleotide polymorphisms (SNPs) and haplotypes of antiplatelet drug metabolism-related genes—*CYP2C19*, *PTGS1*, *ITGB3*, *PEAR1*, and *GP1BA*—as well as their gene-gene (*G* × *G*) and gene-environment (*G* × *E*) interactions on the risk of premature coronary artery disease (PCAD).

**Methods:**

This real-world study enrolled 1,108 participants, including PCAD patients and angiography-referred controls without obstructive CAD. Twelve SNP loci were genotyped using PCR amplification followed by digoxigenin-based colorimetric detection, including *CYP2C19* (rs4244285, rs12248560, rs12769205, rs3758580, rs4917623), *PTGS1* (rs1330344, rs3842788, rs5788), *ITGB3* (rs5918), *PEAR1* (rs12041331)*,* and *GP1BA* (rs6065 and rs2243093). Linkage disequilibrium and population-level haplotype frequencies were assessed using Haploview software, and haplotype carrier status used in regression analyses was inferred from haplotype probability outputs. The generalized multifactor dimensionality reduction (GMDR) method was employed to identify *G* × *G* and *G* × *E* interaction models, and logistic regression was used to evaluate their combined effects.

**Results:**

Significant differences in genotype and allele frequencies between PCAD patients and controls were observed for rs12769205, rs3758580, rs4917623 and rs1330344 (*P* < 0.05). The dominant model of rs12769205 exhibited a protective effect, whereas the dominant models of rs3758580, rs4917623, and rs1330344 were associated with increased PCAD risk (*P* < 0.05). Haplotype analysis showed that *CYP2C19 G-C-C-A-A* and *CYP2C19 G-C-C-C-C* were protective, while *CYP2C19 G-C-A-A-A*, *G-C-A-C-C*, *A-C-C-A-A*, *PTGS1 C-A-A*, *C-A-C*, and *ITGB3-GP1BA A-C-C* increased PCAD risk (*P* < 0.05). GMDR analysis identified significant interaction models. For SNP-SNP interactions, individuals carrying the rs12769205 AC/CC genotype combined with the rs3758580 CC genotype (OR = 0.76, 95% CI: 0.55–0.96, *P* < 0.05), rs12769205 AC/CC genotype without diabetes mellitus (DM) (OR = 0.55, 95% CI: 0.21–0.88, *P* < 0.05), *CYP2C19-C2 (G-C-C-A-A)* carriers without *ITGB3-GP1BA-A2 (A-C-C)* carriage (OR = 0.64, 95% CI: 0.58–0.89, *P* < 0.05), and *CYP2C19-C2* carriers without DM (OR = 0.77, 95% CI: 0.54–0.96, *P* < 0.05) exhibited the lowest PCAD risk. These interaction findings should be interpreted cautiously because they were not externally replicated.

**Conclusion:**

Polymorphisms, haplotypes and interactions of antiplatelet drug-related genes significantly influence the risk of PCAD. Environmental exposures should be considered in early warning and individualized prevention of PCAD.

## Introduction

Coronary artery disease (CAD) is a cardiac disorder caused by myocardial ischemia, hypoxia, or necrosis resulting from luminal narrowing or occlusion due to coronary atherosclerosis. It remains one of the leading causes of disability and mortality worldwide (1). According to the 2023 China Cardiovascular Health and Disease Report, there are currently approximately 330 million patients with cardiovascular disease in China, including 245 million with hypertension, 13 million with stroke, 11.39 million with CAD, and 8.9 million with heart failure. Cardiovascular disease ranks as the leading cause of death among both urban and rural residents (2). With the aging of the population, changes in lifestyle, and the widespread prevalence of risk factors such as hypertension, hyperlipidemia, diabetes, obesity, and smoking, the morbidity and mortality rates of CAD have remained persistently high, imposing a substantial economic burden and health threat on society and families (3). In recent years, the incidence of CAD has shown a trend toward an earlier age of onset. CAD presenting at a younger age is termed premature coronary artery disease (PCAD), typically defined as onset before 55 years of age in men and before 65 years of age in women (4). Studies have indicated that the primary risk factors for PCAD include thrombosis (e.g., smoking, platelet hyperreactivity) (5), atherosclerosis (e.g., hypertension, diabetes, dyslipidemia) (6), and genetic factors (e.g., familial hypercholesterolemia) (7). Therefore, early screening and identification of risk factors in young patients with CAD are essential. An in-depth investigation into the genetic susceptibility factors for PCAD and the search for potential biomarkers are of great significance for elucidating its pathogenesis and achieving early detection and intervention.

Premature coronary artery disease is characterized as an inflammatory condition. Its danger lies in the abundance of vulnerable plaques rich in inflammatory cells, which are prone to rupture. Rupture rapidly triggers platelet aggregation, leading to coronary occlusion. Due to these characteristics, the clinical onset is often acute, frequently presenting as an acute myocardial infarction upon hospital admission. Delayed diagnosis and treatment can result in extensive myocardial necrosis, severe malignant arrhythmias, or cardiogenic shock. These conditions contribute to a higher mortality rate compared to typical CAD (8). Based on the pathogenesis of PCAD, platelet hyperreactivity is often considered one of the causative factors (5).

With the advancement of pharmacogenomics, an increasing number of studies have focused on the relationship between genetic variations and drug response. Clopidogrel and aspirin are antiplatelet drugs commonly used in clinical practice. Clopidogrel is an inactive prodrug. Following oral administration, over half of the administered dose may be absorbed across the intestinal epithelium, whereas *P*-glycoprotein/ABCB1 acts as an efflux transporter that can limit bioavailability by transporting part of the drug back into the intestinal lumen rather than facilitating absorption. After absorption into the bloodstream, approximately 85% of clopidogrel is converted by esterases into an inactive carboxyl metabolite with an 8-hour half-life, while about 15% is converted *in vivo* into an active metabolite via the cytochrome P450 (CYP450) enzyme system (9). This pathway involves two metabolic steps: first, an oxidation reaction generates 2-oxo-clopidogrel, catalyzed by enzymes such as *CYP2C19*, *CYP1A2*, and *CYP2B6* (9, 10); second, a hydrolysis reaction produces the active thiol metabolite, catalyzed by enzymes including *CYP3A4/5*, *CYP2B6*, *CYP2C19*, and *CYP2C9* (10, 11). Cytochrome P450, encoded by the CYP supergene family, is a crucial drug-metabolizing enzyme system in the human liver, playing a determinant role in the metabolism of numerous endogenous and exogenous compounds. Its activity directly influences the rate of drug metabolism (11). The CYP system comprises multiple families and subfamilies. *CYP2C19*, a significant drug-metabolizing enzyme within the CYP450 family, affects the metabolism of many clinically important drugs (12). Mutations in the *CYP2C9* and *CYP2C19* genes can lead to fundamental alterations in the spatial structure and function of the *CYP2C9* and *CYP2C19* enzymes, resulting in decreased or even loss of enzyme activity. This ultimately leads to significant changes in the metabolic clearance capacity of their substrates (13). Furthermore, genes such as *GPIIIa*, *PTGS1* (14), *ITGB3* (15), *PEAR1* (16), and *GP1BA* (16, 17) significantly influence aspirin metabolism.

In recent years, a growing body of research has confirmed that polymorphisms in *CYP2C19*, *CYP2C9*, *PTGS1*, *ITGB3*, *PEAR1*, and *GP1BA* may influence the metabolism, pharmacodynamic response, or clinical efficacy of several cardiovascular and antiplatelet drugs. For example, *CYP2C9* gene polymorphisms affect the metabolism of warfarin (18), diuretics, antiplatelet drugs (19) and selected statins (20), particularly fluvastatin and, to a lesser extent, rosuvastatin, whereas most lipophilic statins are mainly handled by other pathways and transporters rather than *CYP2C9* alone. *CYP2C19* gene polymorphisms influence the metabolism of proton pump inhibitors, benzodiazepines, and antiplatelet drugs (12). Polymorphisms in *PTGS1* (21), *ITGB3*, *PEAR1*, and *GP1BA* genes impact aspirin-related platelet response (14). The effects mediated by these genes can lead to interindividual differences in platelet reactivity and antiplatelet responsiveness following interventional procedures, thereby influencing cardiovascular risk.

Although numerous studies have investigated the relationship between antiplatelet drug-related gene polymorphisms and CAD, most are based on small sample sizes, clinical trials, or specific populations, lacking representativeness and generalizability. Additionally, patients with premature CAD represent a distinct population whose genetic background may differ from those with late-onset CAD. Currently, research on antiplatelet gene polymorphisms specifically targeting this population remains relatively scarce.

This study, based on real-world data, aimed to investigate the impact of 12 core single nucleotide polymorphisms (SNPs) in antiplatelet pathways—including *CYP2C19* (rs4244285, rs12248560, rs12769205, rs3758580 and rs4917623), *PTGS1* (rs1330344, rs3842788 and rs5788), *ITGB3* (rs5918), *PEAR1* (rs12041331), and *GP1BA* (rs6065 and rs2243093*)*—as well as their haplotypes and interactions on the pathogenesis of PCAD. Furthermore, we employed the Multifactor Dimensionality Reduction (MDR) method to conduct association analyses based on haplotype clusters, gene-gene (*G* × *G*) interactions, and gene-environment (*G* × *E*) interactions within this population. The 12 SNPs were selected *a priori* because they are located in genes with established roles in clopidogrel bioactivation, aspirin response, and platelet adhesion/aggregation, and have been reported in prior pharmacogenomic or cardiovascular association studies; the selected loci also have sufficient minor allele frequencies in East Asian or Chinese populations to support case-control association analysis. This selection strategy was intended to balance biological plausibility, prior evidence, and analytical feasibility in a PCAD cohort, rather than to provide comprehensive genome-wide coverage.

## Methods

### Study population

A total of 1,108 participants (634 males, 57.22%; 474 females, 42.78%) were enrolled from patients attending Liuzhou People's Hospital. The mean age was 51.14 ± 7.4 years in the control group and 50.49 ± 6.42 years in the PCAD group.

### Inclusion criteria for PCAD group

1. Age ≥18 years; males <55 years, females <65 years.
2. Diagnosed with coronary artery disease (CAD) by coronary angiography, defined as stenosis >50% in major coronary artery branches.
3. Able to understand the purpose of this study, voluntarily participate and sign the informed consent form, with good post-discharge adherence to medication and willingness to accept clinical follow-up.

### Exclusion criteria

1. Major physical disability or mental illness.
2. Poor adherence or inability to participate in regular follow-up examinations.
3. Pregnancy or planned pregnancy within six months.
4. Considered unsuitable for participation in this study by the investigator.

### Control group

The control group consisted of individuals who underwent coronary angiography during the same period but did not meet the diagnostic criteria for obstructive CAD (stenosis <50% in all major coronary artery branches) and did not meet the exclusion criteria. Therefore, these controls should be interpreted as clinically referred angiography controls rather than completely healthy community controls. This design reduced diagnostic misclassification of CAD status but may introduce ascertainment bias and may limit the generalizability of the findings to the general population.

### Epidemiological investigation

Epidemiological data were collected using standardized international methods. A uniform questionnaire was employed to gather information on demographics, socioeconomic status, and lifestyle factors. Smoking status was categorized as: non-smoker, ≤20 cigarettes/day, or >20 cigarettes/day. Alcohol consumption was categorized as: non-drinker, ≤25 g/day, or >25 g/day. Blood pressure, height, and weight were measured, and body mass index (BMI, kg/m^2^) was calculated.

### Biochemical measurements

Venous blood samples were collected from all participants after a minimum 12-hour fast. Serum levels of total cholesterol (TC), triglycerides (TG), high density lipoprotein cholesterol (HDL-C), and low-density lipoprotein cholesterol (LDL-C) were determined using enzymatic kits (RANDOX Laboratories, Crumlin, UK; Daiichi Pure Chemicals, Tokyo, Japan). Apolipoprotein A1 (ApoA1) and apolipoprotein B (ApoB) were measured using an immunoturbidimetric method (RANDOX). All biochemical analyses were performed using a Hitachi 7170A automatic biochemical analyzer.

### Genotyping

Genomic DNA was extracted from leukocytes using the phenol-chloroform method. Twelve SNP loci were detected using PCR amplification followed by digoxigenin-based colorimetric detection. Amplification and colorimetric reactions were performed using a Tianlong Fascan 48E multichannel fluorescence quantitative analyzer. To clarify assay reliability, genotyping quality control included negative controls, repeated testing of a subset of samples, review of genotype clusters/call signals, exclusion of samples or SNPs with low call rates, and Hardy-Weinberg equilibrium evaluation in the control group. SNPs that failed prespecified quality-control thresholds were not included in the final association analyses.

### Statistical analysis

Statistical analyses were performed using SPSS software (version 26.0). Quantitative data with a normal distribution were expressed as mean ± standard deviation. Triglyceride (TG) levels, which were not normally distributed, were expressed as median (interquartile range, IQR). Comparison of general characteristics between the two groups was performed using analysis of covariance (ANCOVA). Differences in the frequency distributions of genotypes, alleles, haplotypes, and gene-gene (*G* × *G*) interactions between groups were assessed using the *χ*^2^ test.

Hardy-Weinberg equilibrium (HWE), pairwise linkage disequilibrium (LD), and population-level haplotype frequencies were calculated using Haploview software (version 4.2). LD was expressed using *D*′. Because Haploview mainly estimates LD structure and haplotype frequencies, the procedure used to assign individual-level haplotype carrier status for logistic regression should be explicitly verified by the authors. In the revised analysis plan, haplotype carrier status was treated as an inferred variable based on posterior haplotype probabilities and low-confidence assignments were not used for carrier-based sensitivity analyses. Associations of genotypes, haplotypes, and *G* × *G* interactions with PCAD were analyzed using univariate analysis. Multiple testing across SNP, allele, haplotype, and interaction analyses was addressed using false-discovery-rate (FDR) control, while the Bonferroni threshold of *P* < 0.004(0.05/12) was retained only for exploratory lipid-phenotype comparisons involving the 12 SNPs.

Multivariable unconditional logistic regression was used to evaluate the associations of genotypes, alleles, haplotypes, and *G* × *G* interactions with PCAD, after adjusting for age, sex, BMI, blood pressure, smoking, alcohol consumption, and fasting blood glucose. Because the PCAD association analyses involved multiple SNPs, haplotypes, and interaction models, nominal *P* values were interpreted together with multiple-testing considerations, including false discovery rate (FDR) control for exploratory association results. Interaction findings were therefore regarded as hypothesis-generating unless supported by biological plausibility and consistency across models.

The generalized multifactor dimensionality reduction (GMDR) method was employed to identify the optimal combination of interactions among genes, SNPs, and environmental exposures. The cross-validation consistency (CVC) score reflected model stability, and the testing balanced accuracy (Testing Bal.Acc) assessed predictive power, with values ranging from 0.50 to 1.00. Model significance was evaluated using permutation tests (1,000 iterations). The case-control ratio was approximately 1.5:1 (670 PCAD cases and 438 controls). This imbalance reflected consecutive real-world recruitment during the study period; no matched subsample was used, and adjusted regression models were applied to reduce confounding by measured covariates. However, this imbalance may affect the stability of logistic regression estimates, particularly for low-frequency haplotypes.

## Results

### Population characteristics

The epidemiological and clinical indicators of the 1108 study participants are summarized in Table 1. Compared with the non-PCAD group, the PCAD group exhibited significantly higher body mass index (BMI); higher percentages of smoking and alcohol consumption; higher levels of diastolic blood pressure (DBP), pulse pressure (PP), serum glucose, total cholesterol (TC), triglycerides (TG), low-density lipoprotein cholesterol (LDL-C), apolipoprotein B (ApoB), alanine aminotransferase (ALT), aspartate aminotransferase (AST), and uric acid (UA); higher prevalence rates of hypertension, hyperlipidemia, and diabetes mellitus; and higher usage rates of statins, RAAS inhibitors, *β*-blockers, aldosterone receptor antagonists, aspirin, clopidogrel, and ticagrelor. Conversely, the PCAD group showed significantly lower levels of serum high-density lipoprotein cholesterol (HDL-C), serum apolipoprotein A1 (ApoA1), and the ApoA1/ApoB ratio (*P* < 0.05 to *P* < 0.001). No statistically significant differences were observed between the two groups in terms of age, height, systolic blood pressure (SBP), or ejection fraction (EF) (*P* > 0.05 for all indicators).

**Table 1:** A real-world study to explore the impact of antiplatelet gene polymorphisms on early-onset coronary artery disease and selection of therapeutic strategies*.*

Parameter	Control	PCAD	*test-statistic*	*P*
Number	438	670		
Male/female	168/270	466/204	105.3	<0.001
Age (years)	51.14 ± 7.4	50.49 ± 6.42	1.550	0.121
Height (cm)	163.70 ± 7.93	163.93 ± 7.89	0.473	0.636
Weight (kg)	62.60 ± 11.36	67.32 ± 13.56	6.023	<0.001
Body mass index (kg/m^2^)	24.38 ± 3.978	25.47 ± 4.899	3.896	<0.001
Smoking status				
Non-smoker	334 (76.26)	48 (7.16)	559.8	<0.001
≤20 cigarettes/day	51 (11.64)	321 (47.91)		
>20 cigarettes/day	53 (7.91)	301 (44.93)		
Alcohol consumption				
Non-drinker	391 (89.27)	493 (73.58)	40.51	<0.001
≤25 g/day	31 (7.08)	122 (18.21)		
>25 g/day	16 (3.65)	55 (8.21)		
Systolic blood pressure (mmHg)	131.80 ± 18.70	133.20 ± 19.65	1.178	0.2391
Diastolic blood pressure (mmHg)	76.15 ± 11.44	78.51 ± 12.82	3.120	0.0019
Pulse pressure (mmHg)	55.70 ± 13.93	54.74 ± 13.42	27.96	0.0068
Glucose (mmol/L)	7.52 ± 2.66	8.29 ± 4.04	3.532	<0.001
Total cholesterol (mmol/L)	4.43 ± 0.99	4.75 ± 1.09	4.866	<0.001
Triglyceride (mmol/L)	1.63 ± 1.06	2.34 ± 2.03	6.671	<0.001
HDL-C (mmol/L)	1.22 ± 0.34	1.05 ± 0.27	9.092	<0.001
LDL-C (mmol/L)	2.83 ± 0.91	3.15 ± 1.07	5.226	<0.001
ApoA1 (g/L)	1.32 ± 0.25	1.21 ± 0.22	7.731	<0.001
ApoB (g/L)	0.998 ± 0.286	1.073 ± 0.301	4.163	<0.001
ApoA1/ApoB	1.43 ± 0.48	1.23 ± 0.48	6.758	<0.001
ALT	21.93 ± 19.14	30.40 ± 30.78	5.14	<0.001
AST	22.81 ± 36.28	39.16 ± 67.38	4.657	<0.001
T3	1.22 ± 0.22	1.22 ± 0.30	0.01263	0.9899
T4	82.83 ± 16.43	82.96 ± 18.57	0.1155	0.9080
TSH	2.29 ± 2.07	2.392 ± 4.01	0.4927	0.6223
CK	113.3 ± 73.73	131.7 ± 236.9	1.579	0.1153
CKMB	15.63 ± 8.578	19.21 ± 23.44	3.066	0.0022
UA	347.7 ± 101.9	390.9 ± 112.6	6.473	<0.001
Hypertension	132 (30.17)	320 (47.76)	34.06	<0.001
Hyperlipidemia	133 (30.37)	287 (42.84)	17.50	<0.001
DM	43 (9.81)	150 (22.39)	30.75	<0.001
Ato/Rsf	50/9	539/131		
ARNI or ACEI	80 (18.26)	345 (51.49)	123.7	<0.001
BB	44 (10.05)	283 (42.24)	132.0	<0.001
MRA	1 (0.23)	82 (12.24)	55.13	<0.001
Anti-PLT				
Asp	72 (16.44)	573 (85.52)	519.6	<0.001
Clopidogrel	6 (1.37)	224 (33.43)	165.5	<0.001
Ticagrelor	1 (0.23)	96 (14.33)	65.92	<0.001
EF	55.13%	51.515%	0.233	0.587

*HDL-C,* high-density lipoprotein cholesterol; *LDL-C*, low-density lipoprotein cholesterol; *Apo*, Apolipoprotein; *ALT*, Alanine Aminotransferase; *AST*, Aspartate Aminotransferase; *T3*,Triiodothyronine; *T4*, Thyroxine; *TSH*, Thyroid-Stimulating Hormone; *CK*, Creatine Kinase; *CKMB*, Creatine Kinase-MB Isoenzyme; *UA*, Uric Acid; *DM*, Diabetes Mellitus; *Ato*, Atorvastatin; *Rsf*, Rosuvastatin; *ARNI*, Angiotensin Receptor-Neprilysin Inhibitor; *ACEI*, Angiotensin-Converting Enzyme Inhibitor; *BB*, Beta-Blocker; *MRA*, Mineralocorticoid Receptor Antagonist; *Asp*, Aspirin; *EF*, Ejection Fraction.

### Association of SNPs with PCAD

As shown in Table 2, significant differences were observed in the genotype and allele frequencies of the 12 SNP variants between the two groups (*P* < 0.05 to *P* < 0.001). All mutations were in Hardy Weinberg equilibrium (HWE, all *P* > 0.05). The dominant model of the rs12769205 locus exhibited a protective effect, whereas the dominant models of the rs3758580, rs4917623, and rs1330344 loci were associated with an increased risk of disease onset (*P* < 0.05 to *P* < 0.001).

**Table 2:** Association between *CYP2C19*, *PTGS1*, *ITGB3*, *PEAR1*, and *GP1BA* polymorphisms and premature coronary artery disease [n (%)].

Mutation	Gene type	Control (*n* = 438)	PCAD (*n* = 670)	*χ^2^*	*P*-value	OR (95% CI)	*P*-value
*CYP2C19* rs4244285 G > A	GG	307 (70.1)	493 (73.6) (73.6)(73.6%)	1.608	0.2048	1	−
	AG + AA	131 (29.9)	177 (26.4)			1.19 (0.91–1.55)	0.21
	MAF	194 (0.14)	140 (0.16)	0.9362	0.33		
	*P_HWE_*	0.59	0.35				
*CYP2C19* rs1224856° C > A	CC	405 (92.5)	636 (94.9)	2.82	0.0931	1	−
	AC + AA	33 (7.5)	34 (5.1)			1.52 (0.93–2.50)	0.096
	MAF	35 (0.04)	35 (0.03)	3.314	0.0687		
	*P_HWE_*	0.14	0.37				
*CYP2C19* rs12769205 A > C	AA	143 (32.6)	270 (40.3)	6.63	0.01	1	−
	AC + CC	295 (67.3)	400 (59.7)			0.88 (0.46–0.93)	0.0098
	MAF	355 (0.41)	501 (0.37)	2.199	0.1381		
	*P_HWE_*	0.022	0.25				
*CYP2C19* rs3758580 A > C	AA	266 (60.7)	457 (68.2)	6.533	0.0106	1	−
	AC + CC	172 (39.3)	213 (31.8)			1.43 (1.08–1.78)	0.011
	MAF	200 (0.23)	241 (0.18)	7.804	0.0052		
	*P_HWE_*	0.11	0.17				
*CYP2C19* rs4917623 A > C	AA	263 (60)	453 (67.6)	6.632	0.01	1	−
	AC + CC	175 (40)	217 (32.4)			1.45 (1.06–1.98)	0.01
	MAF	203 (0.23)	245 (0.18)	7.853	0.0051		
	*P_HWE_*	0.23	0.15				
*PTGS1* rs1330344 A > C	AA	183 (41.8)	321 (47.9)	4.013	0.0451	1	−
	AC + CC	255 (58.2)	349 (52.1)			1.28 (1.01–1.63)	0.045
	MAF	307 (0.35)	421 (0.31)	3.16	0.0755		
	*P_HWE_*	0.75	0.28				
*PTGS1* rs3842788 A > G	AA	338 (77.2)	491 (73.3)	2.122	0.1452	1	−
	AG + GG	100 (22.8)	179 (26.7)			0.81 (0.61–1.08)	0.14
	MAF	107 (0.12)	189 (0.14)	1.635	0.201		
	*P_HWE_*	0.82	0.34				
*PTGS1* rs5788 A > C	AA	119 (27.2)	168 (25.1)	0.6053	0.4366	1	−
	AC + CC	319 (72.8)	502 (74.9)			0.90 (0.68–1.18)	0.44
	MAF	432 (0.49)	659 (0.49)	0.003918	0.9501		
	*P_HWE_*	0.22	0.49				
*ITGB3* rs5918 A > C	AA	394 (90)	584 (87.2)	1.991	0.1582	1	−
	AC + CC	44 (10)	86 (12.8)			0.76 (0.52–1.11)	0.15
	MAF	46 (0.05)	90 (0.07)	1.974	0.16		
	*P_HWE_*	0.33	0.53				
*PEARl* rs12041331 G > A	GG	159 (36.3)	230 (34.3)	0.4526	0.5	1	-
	AG + AA	279 (63.7)	440 (65.7)			0.92 (0.71–1.18)	0.5
	MAF	362 (0.41)	574 (0.43)	0.4961	0.4812		
	*P_HWE_*	0.11	0.083				
*GPIBA* rs6065 G > C	GG	280 (63.9)	429 (64)	0.001217	0.9722	1	-
	CG + CC	158 (36.1)	241 (36`)			1.0 (0.78–1.29)	0.97
	MAF	177 (0.2)	267 (0.2)	0.02594	0.8720		
	*P_HWE_*	1	0.77				
*GPIBA* rs2243093 T > C	TT	125 (28.5)	191 (28.5)	0.0001	0.99	1	-
	CT + CC	313 (71.5)	479 (71.5)			1.0 (0.76–1.30)	0.99
	MAF	414(0.47)	631(0.47)	0.006196	0.9313		
	*P_HWE_*	0.57	0.59				

*CYP2C19*, encodes an enzyme involved in drug metabolism; *PTGS1*, encodes the enzyme for prostaglandin synthesis; *ITGB3*, encodes the integrin beta3 subunit related to platelet aggregation; *PEAR1*, encodes a protein involved in platelet activation and cell adhesion; *GP1BA*, encodes glycoprotein Ib alpha involved in platelet adhesion; *HWE*, Hardy-Weinberg equilibrium; *MAF*, minor allele frequency.

### Linkage disequilibrium and haplotype analysis

**Figure 1:**
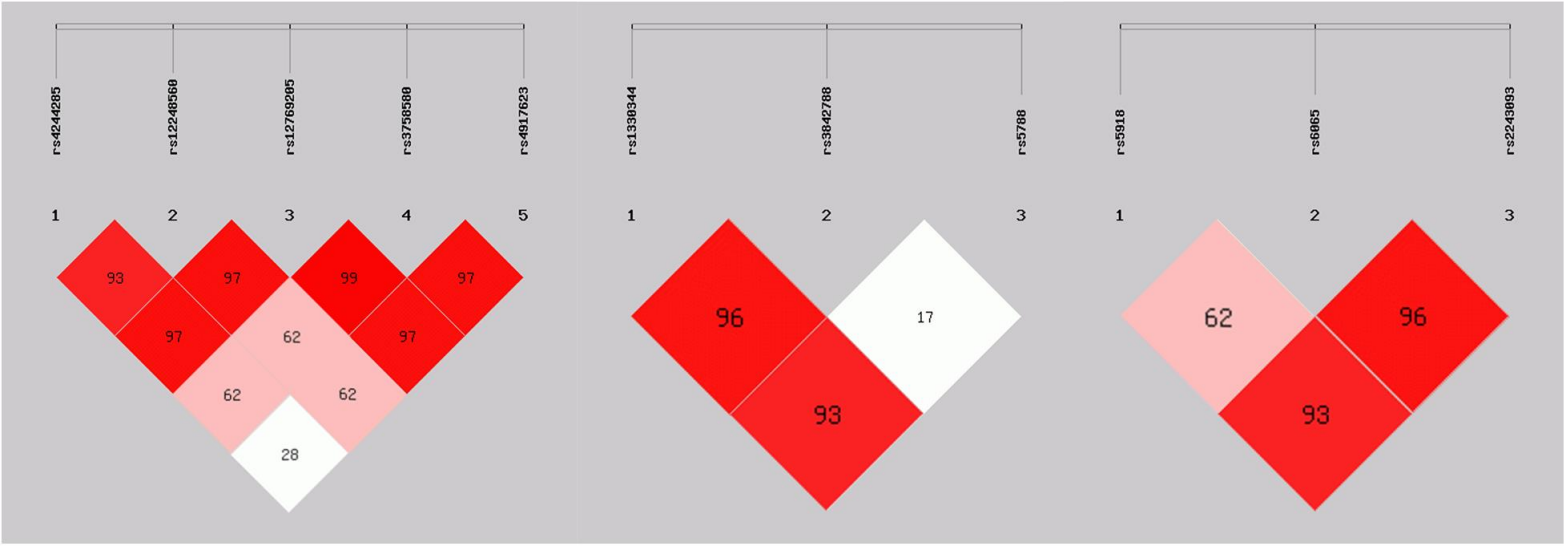
Linkage disequilibrium structure of the *CYP2C19*, *PTGS1*, *ITGB3*, and *GP1BA* loci. The figure displays the LD blocks and combined haplotypes between the two groups.

Multilocus linkage disequilibrium (LD) analysis (Figure 1) revealed that the examined loci were not statistically independent within the study population. Figure 1 displays the LD blocks and combined haplotypes between the two groups.

Haplotype analysis was conducted based on the LD blocks identified. Table 3 presents the haplotype frequencies and their associations with PCAD. The most common haplotypes were *CYP2C19* GCAAA, *PTGS1* AAA, and *ITGB3GP1BA* AGT, each with a frequency >30%. Significant differences in haplotype frequencies between the PCAD and control groups were observed for the following haplotypes: *CYP2C19 G-C-A-A-A*, *CYP2C19 G-C-C-A-A*, *CYP2C19 G-C-A-C-C*, *CYP2C19 G-C-C-C-C*, *CYP2C19 A-C-C-A-A*, *PTGS1 C-A-A*, *PTGS1 C-A-C,* and *ITGB3-GP1BA A-C-C.*

**Table 3:** Haplotype frequencies and their associations with premature coronary artery disease [n (frequency)].

NO.	Haplotype	PCAD	Control	χ^2^	*P*-value	Odd Ratio [95% CI]
C1	*CYP2C19 G-C-A-A-A*	275.24 (0.4108)	155.97 (0.3561)	3.284	0.024	1.22 (1.08–1.87)
C2	*CYP2C19 G-C-C-A-A*	171.92 (0.2566)	108.41 (0.2475)	0.1442	0.036	0.86 (0.74–0.94)
C3	*CYP2C19 G-C-A-C-C*	60.17 (0.0898)	50.02 (0.1142)	1.793	0.004	1.54 (1.04–2.29)
C4	*CYP2C19 A-C-A-A-A*	56.28 (0.084)	32.28 (0.0737)	0.4011	0.102	1.02 (0.65–1.61)
C5	*CYP2C19 G-C-C-C-C*	36.78 (0.0549)	32.98 (0.0753)	62.45	0.019	0.40 (0.27–0.85)
C6	*CYP2C19 A-C-C-A-A*	22.45 (0.0335)	20.85 (0.0476)	1.621	0.013	1.67 (0.87–3.19)
P1	*PTGS1* A-A-A	204.41 (0.3051)	132.143 (0.3017)	0.01211	0.163	0.77 (0.26–4.27)
P2	*PTGS1* A-A-C	192.36 (0.2871)	116.07 (0.265)	0.6229	0.085	0.94 (0.71–1.25)
P3	*PTGS1* C-A-A	96.82 (0.1445)	67.76 (0.1547)	0.2293	0.074	1.10 (0.77–1.57)
P4	*PTGS1* C-A-C	81.94 (0.1223)	68.50 (0.1564)	2.779	0.013	1.27 (1.15–1.70)
P5	*PTGS1* A-G-C	41.88 (0.0625)	22.86 (0.0522)	0.4966	0.063	0.85 (0.52–1.38)
P6	*PTGS1* A-G-A	20.90 (0.0312)	13.40 (0.0306)	0.02462	0.1225	0.99 (0.49–2.00)
A1	*ITGB3*-GPIBA A-G-T	337.61 (0.5039)	220.71 (0.5039)	0.202	0.63	1.06 (0.92–1.28)
A2	*ITGB3*-GPIBA A-C-C	166.96 (0.2492)	108.97 (0.2488)	2.425	0.006	1.21 (1.08–1.83)
A3	*ITGB3*-GPIBA A-C-C	119.06 (0.1777)	83.658 (0.1916)	0.3553	0.091	1.07 (0.85–1.36)

Haplotypes were constructed from *CYP2C19* (rs4244285, rs12248560, rs12769205, rs3758580 and rs4917623), *PTGS1* (rs1330344, rs3842788 and rs5788), *ITGB3* (rs5918), and *GP1BA* (rs6065 and rs2243093). Frequencies less than 0.3% were excluded from the statistical analysis.

The *CYP2C19 G-C-C-A-A* and *CYP2C19 G-C-C-C-C* haplotypes exhibited a protective effect against PCAD. In contrast, the *CYP2C19 G-C-A-A-A*, *CYP2C19 G-C-A-C-C*, *CYP2C19 A-C-C-A-A*, *PTGS1 C-A-A*, *PTGS1 C-A-C*, and *ITGB3-GP1BA A-C-C* haplotypes were associated with an increased risk of disease onset (*P* < 0.05 to *P* < 0.001).

### Gene-Gene and gene-environment interaction analysis

**Figure 2:**
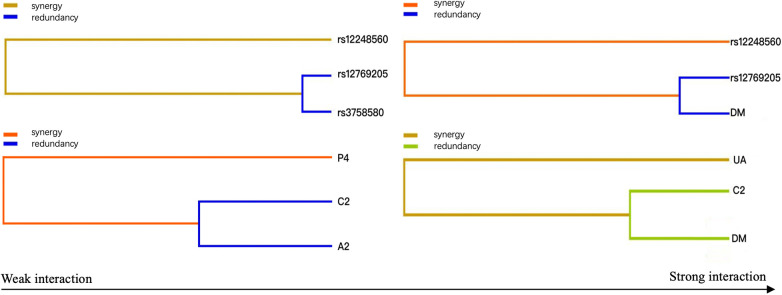
Entropy based interaction dendrogram. The dendrogram reveals that rs12248560 and rs12769205 exhibited the strongest synergistic effect in SNP-SNP interactions. However, redundant effects were observed in the SNP-SNP interaction involving rs3758580 and rs12248560, the haplotype-haplotype interactions (DM-C2-A2 and P4-UA-C2), and the haplotype-environment interactions (C2-A2-DM and P4-UA-C2).

The generalized multifactor dimensionality reduction (GMDR) model was employed to evaluate the impact of gene-gene and gene-environment interactions on PCAD. The entropy-based interaction dendrogram (Figure 2) revealed that rs12248560 and rs12769205 exhibited the strongest synergistic effect in SNP-SNP interactions. However, redundant effects were observed in the SNP-SNP interaction involving rs3758580 and rs12248560, the haplotype-haplotype interactions (DM-C2-A2 and P4-UA-C2), and the haplotype-environment interactions (C2-A2-DM and P4-UA-C2).

The most significant two locus SNP-SNP model (*P* < 0.001) involved the rs12769205 and rs3758580 SNPs, with a cross-validation consistency (CVC) of 10/10 and a testing accuracy of 70.82%. The most significant three locus model (*P* < 0.001) involved the rs12769205, rs3758580, and rs12248560 SNPs, with a CVC of 10/10 and a testing accuracy of 79.91%.

For SNP-environment interactions, the most significant two locus model (*P* < 0.001) involved the rs12769205 SNP and diabetes mellitus (DM), with a CVC of 9/10 and a testing accuracy of 71.45%. The most significant three locus model (*P* < 0.001) involved the rs12769205 and rs12248560 SNPs and DM, with a CVC of 10/10 and a testing accuracy of 78.41%.

For haplotype-haplotype interactions, the most significant two-locus model (*P* < 0.001) involved *CYP2C19-C2 (G-C-C-A-A)* and *ITGB3-GP1BA-A2 (A-C-C)*, with a CVC of 10/10 and a testing accuracy of 71.33%. The most significant three-locus model (*P* < 0.001) involved *CYP2C19-C2*, *ITGB3-GP1BA-A2*, and *PTGS1-P4 (C-A-C)*, with a CVC of 10/10 and a testing accuracy of 66.25%.

For haplotype-environment interactions, the most significant two-locus model (*P* < 0.001) involved *CYP2C19-C2 (G-C-C-A-A)* and DM, with a CVC of 9/10 and a testing accuracy of 70.64%. The most significant three-locus model (*P* < 0.001) involved *CYP2C19-C2*, DM, and uric acid (UA), with a CVC of 10/10 and a testing accuracy of 67.74%. The GMDR results for all interaction models are summarized in Table 4.

**Table 4:** GMDR analysis of SNP-SNP, SNP-environment, haplotype-haplotype, and haplotype-environment interactions.

Locus no.	Best combination	Training Bal. Acc	Testing Bal. Acc	Cross-validation consistency	*P*	*P* *
SNP-SNP interactions						
2	rs12769205 rs3758580	0.7341	0.7082	10/10	<0.001	<0.001
3	rs12769205 rs3758580 rs12248560	0.8558	0.7991	10/10	<0.001	<0.001
SNP-environment interactions						
2	rs12769205 DM	0.7644	0.7145	9/10	<0.001	<0.001
3	rs12769205 rs12248560 DM	0.8043	0.7841	10/10	<0.001	<0.001
Haplotype-haplotype interactions						
2	C2 A2	0.7345	0.7133	10/10	0.0023	0.0018
3	C2 A2 P4	0.7069	0.6625	10/10	<0.001	<0.001
Haplotype-environment interactions						
2	C2 DM	0.7201	0.7064	9/10	<0.001	<0.001
3	C2 DM UA	0.6937	0.6774	10/10	<0.001	<0.001

* Indicates 1,000 permutation tests. C2 = *CYP2C19 G-C-C-A-A*; A2 = *ITGB3-GP1BA A-C-C*; P4 = *PTGS1 C-A-C*; *DM* = diabetes mellitus; *UA* = uric acid.

Logistic regression analysis was performed to obtain the odds ratios (OR) and 95% confidence intervals (CI) for the interaction models (Table 5).

**Table 5:** Logistic regression analysis of different interaction models.

Variable 1	Variable 2	OR (95% CI)	*P*-value
SNP-snp interactions			
rs12769205	rs3758580		
AA	AA	1	—
CC	AC + CC	1.89 (1.76–2.32)	0.0623
AC + CC	CC	0.76 (0.55–0.96)	0.0142
AC + CC	AC + CC	0.84 (0.72–0.98)	1.7 × 10^−4^
SNP-environment interactions			
rs12769205	DM		
AA	No	1	—
CC	Yes	0.88 (0.58–0.92)	0.053
AC + CC	No	0.55 (0.21–0.88)	0.041
AC + CC	Yes	0.83 (0.54–0.94)	0.0025
Haplotype-haplotype interactions			
C2	A2		
No-carriers	No-carriers	1	—
Carriers	No-carriers	0.64 (0.58–0.89)	0.0011
No-carriers	Carriers	1.61 (1.02–1.88)	0.0678
Carriers	Carriers	0.83 (0.64–0.94)	2.3 × 10^−5^
Haplotype-environment interactions			
C2	DM		
No-carriers	No	1	—
No-carriers	Yes	1.23 (1.02–2.76)	0.271
Carriers	No	0.77 (0.54–0.96)	0.041
Carriers	Yes	0.92 (0.75–0.99)	0.0025

C2 = *CYP2C19 G-C-C-A-A*; A2 = *ITGB3-GP1BA A-C-C*; *DM* = diabetes mellitus.

## Discussion

This single-center real-world cohort study (*n* = 1,108) adds to the limited evidence regarding the genetic architecture of premature coronary artery disease (PCAD) risk in a Chinese Han population, focusing on 12 core SNPs in antiplatelet pathways, their haplotypes, and gene-gene/gene-environment interactions. The main findings are as follows: (1) we elucidated the distribution frequencies of single nucleotide variants, haplotypes, and gene-gene (*G* × *G*) interactions involving the *CYP2C19*, *PTGS1*, *ITGB3*, *PEAR1*, and *GP1BA* genes in the PCAD population; (2) we provided exploratory evidence for single nucleotide variants, haplotypes, *G* × *G*, and gene-environment (*G* × *E*) interactions, suggesting potential interplay between these genes and PCAD; (3) we detected differential effects based on SNP-SNP, SNP-environment, haplotype-haplotype, and haplotype-environment interactions; (4) we found that different interaction patterns may contribute variably to PCAD risk.

This study identified associations between mutations in *CYP2C19*, *PTGS1*, *ITGB3*, *PEAR1*, and *GP1BA* and PCAD, with significant differences in genotype and allele frequencies of the 12 SNPs between the two groups, suggesting that genetic factors may contribute to the prevalence of PCAD. Analysis of the relationship between individual SNPs and PCAD risk revealed that rs12769205 was associated with decreased risk. However, SNP-environment interaction analysis showed that the risk was further reduced in individuals carrying the rs12769205 AC/CC genotype without diabetes mellitus. Similar results were observed for haplotype-haplotype, haplotype-environment, gene-gene, and gene-environment interactions. A plausible interpretation is that both genetic factors and the metabolic environment collectively drive the pathogenesis of this premature form of coronary artery disease.

In recent years, pharmacogenomic research on antiplatelet drugs has been evolving from single-locus analysis towards the dissection of polygenic synergy and interaction effects. An integrated pharmacogenomic analysis published in 2025 indicated that interactions between polymorphisms in PON1 (rs662), *ABCB1* (rs1045642), *P2RY12* receptor genes (rs6809699, rs3732759), and *CYP2C19* collectively influence platelet reactivity, with multi-gene comprehensive assessment models predicting cardiovascular risk more accurately than single-gene testing (22, 23). A growing body of research suggests that the occurrence of CAD is influenced by a multitude of clinical and lifestyle factors (24). The interaction effects observed in this study between protective and risk loci (e.g., rs12769205 and rs3758580) and between protective and risk haplotypes (e.g., C2 and A2) suggest that future PCAD risk assessment should consider multi-gene combined effects rather than single mutations alone. Furthermore, international expert consensus related to the 2024 ACC/AHA guidelines has incorporated pharmacogenomic testing into decision-making references for antiplatelet therapy in specific populations, emphasizing the importance of individualized treatment (25).

In the realm of gene-environment interactions, this study identified a significant phenomenon: the protective genotype (rs12769205 AC/CC) exhibited the strongest protective effect in individuals without DM (OR = 0.55), whereas this protective effect was attenuated in those with DM. This finding suggests that DM may partially counteract the genetic protective effect of the *CYP2C19* rs12769205 variant through mechanisms such as metabolic dysregulation, chronic inflammation, or epigenetic modifications (26). In other words, a favorable metabolic environment is a necessary condition for the full expression of genetic protective factors. When the metabolic environment deteriorates (e.g., in the presence of DM), the capacity of even protective genotypes to reduce PCAD risk may be limited. This “gene-environment antagonism” phenomenon underscores a critical insight: in the prevention and treatment of premature coronary artery disease, controlling metabolic risk factors (such as preventing and managing DM) is equally as important as considering genetic background—genetic advantages cannot fully counteract the pathogenic effects of an adverse metabolic environment. Recent studies suggest significant racial differences in antiplatelet treatment response, with the carrier frequency of *CYP2C19* loss-of-function alleles in East Asian populations reaching 50%–60%, significantly higher than in European and American populations (27). This study validates and extends this understanding within a Chinese Han population, providing genetic epidemiological evidence for establishing PCAD risk prediction models suitable for the Chinese population.

However, this study has several limitations. First, no independent replication cohort was available. Therefore, the SNP, haplotype, and especially GMDR-based *G* × *G*/*G* × *E* interaction findings may be vulnerable to false-positive results and model overfitting, and should be considered hypothesis-generating until externally validated. Second, the sample size was modest for high-order interaction modeling, and the minor allele frequencies of some variants were low, which may have limited statistical power. Third, the control group consisted of angiography-referred individuals with <50% stenosis rather than population-based healthy controls; this may introduce ascertainment bias and restrict the generalizability of the findings. Fourth, cases and controls differed substantially in sex distribution and smoking status. Although regression models adjusted for sex, smoking, and other measured covariates, dedicated subgroup analyses by sex and smoking were not sufficiently powered and should be explored in larger cohorts. Fifth, the case-control ratio was imbalanced (670:438), which may affect estimate stability for low-frequency haplotypes. Sixth, the PCAD group had higher use of statins, antiplatelet agents, RAAS inhibitors, beta-blockers, and other cardiovascular medications. These therapies can influence lipid profiles, metabolic indices, and platelet function, and residual medication-related confounding may have affected the *G* × *E* analyses. Finally, numerous unmeasured environmental and genetic factors, including dietary patterns, physical activity, and energy intake, require further consideration. Higher-quality studies, including external replication and *in vitro* and *in vivo* functional investigations, are needed to confirm the molecular mechanisms of *CYP2C19*, *PTGS1*, *ITGB3*, *PEAR1*, and *GP1BA* variants, haplotypes, and interactions at the transcriptional and expression levels.

## Conclusion

This study suggests potential interactions among *CYP2C19*, *PTGS1*, *ITGB3*, *PEAR1*, and *GP1BA* genes, environmental exposures, and PCAD risk. Association analyses based on haplotype clusters and *G* × *G* interactions may improve the predictive capacity for PCAD risk compared with single-locus testing alone, but these exploratory findings require validation in independent cohorts. GMDR analysis revealed that different gene-environment interaction patterns exhibited varying synergistic or redundant effects on disease risk. Beyond genetic factors, environmental exposures and clinical confounders constitute crucial components that cannot be overlooked. This study provides preliminary genetic epidemiological evidence for primary prevention strategies and individualized antithrombotic treatment in premature coronary artery disease.

## Data Availability

The raw data supporting the conclusions of this article will be made available by the authors, without undue reservation.
